# Editorial

**Published:** 2012-08-16

Dear Friends,

These are exciting times to be living in and very especially for those of us practicing glaucoma. There has been an explosion, in terms of both knowledge of the disease and its practice. Yet, all of us know how little we know, and how little it is that we can actually do for our patients, by way of disability limitation. That, we feel, is the ideal setting for a revolution in the clinical practice of glaucoma. Like all of us, we hope to witness it in our lifetime.

The editorial team at Journal of Current Glaucoma Practice (JoCGP), the official journal of International Society of Glaucoma Surgery, hopes to chronicle these new developments in the field of glaucoma for you. We are indeed grateful to the dynamic and illustrious Editorial Board for agreeing to join us in this endeavour.

In this issue, Skalicky et al discuss the implications of the increasing use of anti-vascular endothelial growth factor agents in glaucoma practice in the perspectives section. Khanna, in a related review, critically evaluates bevacizumab in glaucoma surgeries.

Our experts for this issue, Profs Grehn, Goldberg and Chew, give their opinion on management of post trabeculectomy hypotony. Sharma et al report a case of bleb revision using reversed scleral flap and pedical conjunctival graft in a case of persistent hypotony after trabeculectomy.

Li et al review the clinical implications of anterior segment optical coherence tomography in glaucoma, while the assessment of structural glaucoma progression is dealt within the diagnostics section by Miki.

Jha et al provide a broad overview of selective laser trabeculoplasty in the current glaucoma treatment paradigm. Almodin et al describe their technique of blitz anesthesia in non-English-speaking patients undergoing glaucoma surgery.

We look forward to the challenges that are ahead of us and will always welcome any comments, criticisms or suggestions that you may have.

Sincerely

**Editors**

